# Serum level of IL-1ra was associated with the treatment of latent tuberculosis infection in a Chinese population

**DOI:** 10.1186/s12879-020-05047-x

**Published:** 2020-05-08

**Authors:** Haoran Zhang, Xuefang Cao, Henan Xin, Jianmin Liu, Shouguo Pan, Ling Guan, Fei Shen, Zisen Liu, Dakuan Wang, Xueling Guan, Jiaoxia Yan, Boxuan Feng, Na Li, Qi Jin, Lei Gao

**Affiliations:** 1 NHC Key Laboratory of Systems Biology of Pathogens, Institute of Pathogen Biology, and Center for Tuberculosis Research, Chinese Academy of Medical Sciences and Peking Union Medical College, No. 9 Dong Dan San Tiao, Dongcheng District, Beijing, 100730 China; 2 The Sixth People’s Hospital of Zhengzhou, Zhengzhou, 450061 China; 3 The Center for Disease Prevention and Control of Zhongmu County, Zhengzhou, 451470 China; 4 Gastroenterology Department, PLA Rocket Force Characteristic Medical Center, Beijing, 100088 China

**Keywords:** Latent tuberculosis infection, Preventive treatment, Cytokines, IL-1ra, Disease development

## Abstract

**Background:**

Dynamically changed levels of serum cytokines might predict the development of active TB from latent tuberculosis infection (LTBI) and monitor preventive treatment effectiveness. The aim of the study was to identify potential serum cytokines associated with LTBI treatment which might predict active disease development in a Chinese population.

**Methods:**

Based on a randomized controlled trial aiming to explore short-course regimens for LTBI treatment, the dynamic changes of serum cytokines determined by bead-based multiplex assays were investigated for the participants who developed active TB during follow-up and age and gender matched controls stayed healthy.

**Results:**

Totally, 21 patients diagnosed with active tuberculosis (TB) during the 2-year follow-up (12 from treated groups and 9 from untreated controls) and 42 age and gender matched healthy controls (24 from treated groups and 18 from untreated controls) were included in the study. Before treatment, serum IL-1ra was statistically higher among those who developed active disease during follow-up as compared with those stayed healthy. As for treated participants, the levels of IL-1ra were significantly lower after treatment in comparison with those before treatment both in active TB group (*p* = 0.002) and non-TB group (*p* = 0.009). For untreated participants, the levels of IL-1ra were not statistically different between different time points both in active TB group (*p* = 0.078) and non-TB group (*p* = 0.265).

**Conclusion:**

Our results suggested that declined serum level of IL-1ra was associated with LTBI treatment. Further studies are needed to verify whether it could be used to evaluate LTBI treatment and to predict active disease development.

## Background

About a quarter of the world’s population were estimated to be latently infected with *Mycobacterium tuberculosis* (*M.tb*) [1]. It was estimated that 5–10% infections might develop active tuberculosis (TB) during their lifetime [2, 3]. Scaling up latent tuberculosis infection (LTBI) testing and treatment among individuals at high-risk of developing active disease is a critical priority action for the END TB [4–6]. Currently, directly observe the decline of incidence is the standard way to evaluate the protective effect of the preventive treatment, which usually needs a long follow-up period and huge resource input. Tuberculin Skin Test (TST) and Interferon-γ Release Assays (IGRAs) both are immunological tests to identify infections, but they are poor at predicting the development of active disease [7, 8]. Accessible surrogate biomarkers could reflect the effectiveness of LTBI treatment are warranted in the era of prevention centered.

It has been recognized that cytokines and chemokines play important roles in shaping immunity against TB by polarizing T cell subsets responses, modulating immune cell trafficking, and regulating inflammatory responses [9]. Some exploratory studies evaluated specific antigen stimulated or un-stimulated serum cytokine biomarkers other than IFN-γ for monitoring the potential effect of anti-TB treatment, such as TNF-α, IL-10, IL-6, IL-1ra, MIP-1β, IL-2/IFN-γ and IP-10 [10–14]. Nevertheless, few studies have been conducted to assess the performance of un-stimulated serum cytokine levels in monitoring host response to preventive treatment of LTBI [12]. Therefore, the aim of the present study was to identify potential cytokine biomarkers associated with prophylactic treatment which might also predict the development of TB from LTBI.

## Methods

### Study population

Study participants in current study were selected from a randomized controlled trial (RCT) exploring short-course treatment regimen for 50–70 years rural residents with LTBI in China. Detailed information of the trial has been published elsewhere [6]. Briefly, all participants aged 50 to 70 years old with QuantiFERON TB Gold In-Tube (QFT, Qiagen, USA) positive result (TB Ag-Nil ≥0.35 IU/ml) and without current active TB at baseline survey were included for a RCT. They were randomly classified into three groups (Group A: 8 weeks regimen of once-weekly RPT plus INH, between 7 November 2015 and 2 January 2016; Group B: 6 weeks of twice-weekly RPT plus INH, between 25 November 2015 and 2 January 2016; Group C: untreated controls). During the 2-year follow-up after the preventive treatment, a total of 30 incident cases of active TB were diagnosed. Among them, 21 active TB cases (Group A = 8; Group B = 4; Group C = 9) with available blood samples and who completed the assigned regimes were included in current study. In addition, 42 gender and age matched non-TB subjects (Group A = 16; Group B = 8; Group C = 18) were included in the present study as well. The protocol of the present study has been approved by the Ethics Committees of the Institute of Pathogen Biology, Chinese Academy of Medical Sciences (IDs: IPB-2015-5 and IPB-2016-8). All participants have signed the written informed consent.

### Cytokines measurements

Blood samples at baseline (T0, 1 week before starting treatment) and at the end of preventive treatment (T1, 1 week after completing the treatment) have been collected and retained. Ready-made cytokine Kit (Bio-Rad Laboratories, Hercules, CA, USA) which could simultaneously detect forty-eight cytokines (CTACK, Eotaxin, GRO-α, interferon-inducible protein (IP)-10/CXCL10, macrophage chemoattractant protein (MCP-1)/MCAF, MCP-3, MIF, MIG, macrophage inflammatory protein-1α (MIP-1α), MIP-1β, PDGF-BB, RANTES, SDF-1α, granulocyte macrophage colony stimulating factor (GM-CSF), G-CSF, SCF, M-CSF, VEGF-A, LIF, Basic FGF, HGF, β-NGF, SCGF-β, IFN-α2, IFN-γ, tumor necrosis factor (TNF)-α, TNF-β, TRAIL, Interleukin (IL)-1α, IL-1β, IL-1ra, IL-2Rα, IL-2, IL-3, IL-4, IL-5, IL-6, IL-7, IL-8, IL-9, IL-10, IL-12(p70), IL-12(p40), IL-13, IL-15, IL-16, IL-17A and IL-18) were used. Levels of the selected cytokines were determined for each undiluted and un-stimulated serum sample (50 μl) by magnetic bead suspension array using the Bio-Plex Pro Human Cytokine panels according to the manufacturer’s instructions. Cytokines with > 50% of the samples below the lower detection level (LDL) of the assay will be excluded from further statistical analysis. Additionally, cytokines with occasional values (< 50%) below the LDL were assigned an averaged value between 0 and the lowest detectable level in each assay plate [15].

### Statistical analysis

Statistical analyses were performed using SAS 9.4 version (SAS institute, Cary, NC) and GraphPad Prism 5 (GraphPad software, San Diego, CA). Participants who completed ≥90% doses of the therapy were defined as completed the regimes. The Chi-square and Fisher’s exact tests were used to compare the distribution of categorical variables across groups. The level of cytokines was presented with median (Q25-Q75). Wilcoxon rank sum test was used to compare cytokine responses between different participants at the same time-point. Wilcoxon signed rank test was used to evaluate the cytokine responses for the same person at different time points. To assess the predictive ability of the cytokines, receiver operating characteristic (ROC) analysis was conducted and the area under ROC curves (AUCs) was calculated. Sensitivities and specificities were also calculated using the value with the highest Youden Index as the cut-off. A two-tailed *p*-value < 0.05 was considered statistically significant.

## Results

### Characteristics of the study participants

Table 1 shows major characteristics of the study participants. For both treated and untreated participants, no significant difference was found between active TB and non-TB groups with respect to gender, age, fasting blood glucose and QFT results. However, active TB patients had lower median body mass index (BMI) level (22.46 kg/m^2^) than non-TB controls (25.53 kg/m^2^) among untreated participants (*p* = 0.029). At baseline, active TB patients showed a trend with higher proportion of IFN-γ values ≥0.70 IU/ml than non-TB controls, but such a difference was not statistically significant.

**Table 1:** Characteristics of the participants included in the study

	Treated participants			Untreated controls		
	Developed active TB during follow-up	Kept healthy during follow-up	*p* value	Developed active TB during follow-up	Kept healthy during follow-up	p value
Total^a^	12	24		9	18	
Median age (Q25-Q75) (years)	69.00 (65.00, 72.50)	69.00 (65.00, 73.00)	0.973^b^	67.00 (62.00, 70.00)	67.00 (62.00, 70.00)	0.979^b^
Gender, n (%)			1.000^c^			1.000^d^
Male	6 (50.00)	12 (50.00)		7 (77.78)	14 (77.78)	
Female	6 (50.00)	12 (50.00)		2 (22.22)	4 (22.22)	
Median BMI (Q25-Q75) (Kg/m^2^)	23.54 (19.67, 26.66)	24.11 (21.71, 27.25)	0.535^b^	22.46 (20.69, 23.88)	25.53 (21.81, 28.00)	0.029^b^
Fasting blood glucose, n (%)			0.253^d^			0.539^d^
≥7.0 mmol/L	2 (16.67)	1 (4.17)		0 (0.00)	2 (11.11)	
<7.0 mmol/L	10 (83.33)	23 (95.83)		9 (100.00)	16 (88.89)	
Median INF-γrelease of QFT (Q25-Q75) (IU/ml)						
T0	2.47 (1.76, 5.06)	2.79 (0.90, 4.81)	0.651^b^	2.06 (1.52, 2.81)	1.50 (0.92, 3.29)	0.520^b^
T1	1.52 (0.91, 3.30)	0.87 (0.18, 1.91)	0.095^b^	0.73 (0.63, 1.06)	0.63 (0.23, 1.51)	0.471^b^
Classified QFT results at T0 (IU/ml), n (%)						
0.35 ~ 0.70	0 (0.00)	4 (16.67)	0.279^d^	1 (11.11)	4 (22.22)	0.636^d^
≥0.70	12 (100.00)	20 (83.33)		8 (88.89)	14 (77.87)	

*Abbreviation*: *BMI* body mass index, *Q25* 25% quantile, *Q75* 75% quantile, *QFT* QuantiFERON-TB Gold In-Tube, *TB* tuberculosis, *T0* baseline, *T1* At the end of preventive treatment. ^a^Data might not sum to total because of missing data

^b^p for Wilcoxon rank sum test. ^c^ p for χ^2^ test. ^d^p for Fisher’s exact test

### Serum cytokine levels in treated and untreated participants at T0

Table 2 showed the baseline median level of cytokines among participants with and without TB occurrence classified by preventive treatment**.** The levels of 10 tested cytokines (LIF, TNF-β, IL-2, IL-3, IL-5, IL-7, IL-10, IL-12(p70), IL-12(p40) and IL-15) were below the LDL, they were not included for further data analysis.

**Table 2:** Median serum levels of selected cytokines among participants with and without TB occurrence classified by preventive treatment at T0 (Median [Q25-Q75] (pg/ml))

Cytokines	Untreated controls			Treated participants		
	Active TB (*n* = 9)	Non-TB (*n* = 18)	P for Wilcoxon rank sum test	Active TB (*n* = 12)	Non-TB (*n* = 24)	P for Wilcoxon rank sum test
CTACK	317.71 (247.15, 392.20)	339.63 (288.62, 381.48)	0.817	245.16 (183.03, 342.38)	340.84 (260.21, 385.66)	0.164
Eotaxin	86.14 (56.31, 100.06)	34.46 (29.40, 37.82)	**< 0.001**	69.18 (58.08, 87.05)	37.14 (26.61, 66.58)	**0.008**
GRO-α	88.03 (74.31, 136.90)	79.17 (69.80, 84.97)	0.227	113.26 (100.91, 130.08)	69.82 (55.58, 89.86)	**< 0.001**
IP-10	564.61 (372.63, 670.62)	373.06 (329.38, 525.68)	0.173	514.05 (380.24, 606.51)	438.45 (351.99, 499.43)	0.275
MCP-1(MCAF)	12.60 (9.20, 16.31)	6.81 (5.14, 9.74)	**0.017**	14.08 (10.57, 20.05)	7.91 (5.65, 10.10)	**< 0.001**
MCP-3	1.15 (0.30, 1.96)	0.83 (0.42, 1.07)	0.738	1.78 (0.45, 3.59)	0.80 (0.43, 1.70)	0.354
MIF	305.23 (227.36, 405.16)	282.69 (255.65, 348.78)	0.396	318.45 (195.43, 358.95)	271.78 (220.06, 312.82)	0.322
MIG	356.29 (308.93, 590.65)	285.33 (179.67, 366.12)	0.129	371.04 (226.06, 612.31)	308.06 (214.66, 428.46)	0.430
MIP-1α	1.35 (1.17, 1.82)	1.18 (0.95, 1.35)	0.100	1.47 (1.41, 1.85)	1.32 (0.94, 1.51)	0.024
MIP-1β	48.10 (38.42, 57.29)	38.03 (33.57, 43.83)	**0.048**	50.75 (47.05, 63.37)	38.06 (30.83, 44.48)	**< 0.001**
PDGF-BB	64.30 (42.51, 198.85)	84.51 (26.05, 114.55)	0.425	146.33 (98.52, 232.85)	43.91 (26.70, 71.55)	**0.001**
RANTES	1314.40 (677.29, 1607.11)	1134.09 (467.87, 1437.52)	0.488	1194.57 (839.53, 2194.69)	762.83 (468.35, 1217.44)	0.050
SDF-1α	268.74 (253.62, 357.07)	219.86 (181.17, 275.33)	**0.017**	309.99 (234.09, 339.68)	211.88 (190.64, 251.72)	**0.008**
SCF	37.10 (27.55, 46.29)	47.10 (32.85, 55.57)	0.456	42.94 (28.96, 50.35)	40.35 (26.53, 56.04)	0.960
M-CSF	33.51 (25.14, 38.47)	35.26 (26.15, 49.94)	0.700	31.64 (22.22,42.16)	37.66 (27.04, 50.64)	0.275
G-CSF	70.18 (68.86, 89.17)	45.96 (39.89, 52.46)	**0.001**	89.49 (73.15, 97.55)	49.40 (36.56, 62.44)	**< 0.001**
GM-CSF	0.79 (0.61, 1.25)	0.60 (0.01, 1.13)	0.255	1.03 (0.70, 2.37)	0.77 (0.14, 1.33)	0.122
VEGF-A	30.46 (19.20, 52.30)	10.62 (3.37, 15.07)	**0.006**	46.18 (38.72, 62.85)	13.34 (3.37, 23.12)	**< 0.001**
Basic FGF	19.51 (19.51, 27.46)	9.91 (7.71, 11.27)	**< 0.001**	23.62 (20.22, 25.25)	11.60 (7.52, 17.13)	**< 0.001**
β-NGF	0.74 (0.38, 0.92)	0.47 (0.15, 0.74)	0.226	0.88 (0.74, 1.51)	0.56 (0.28, 0.74)	**0.002**
SCGF-β	43,169.60 (38,013.20, 57,726.50)	40,842.80 (25,219.40, 45,309.10)	0.316	43,618.70 (38,251.70, 50,595.10)	41,198.40 (34,222.50, 49,973.30)	0.513
HGF	256.86 (249.02,352.46)	227.92 (173.75, 340.78)	0.292	245.12 (213.87, 301.36)	229.34 (160.76, 297.97)	0.557
IFN-α2	5.61 (4.07, 9.52)	4.35 (2.73, 4.90)	0.060	8.14 (6.93, 10.77)	4.71 (2.95, 5.85)	**< 0.001**
IFN-γ	26.08 (18.59,31.97)	9.48 (8.21, 14.39)	**< 0.001**	20.89 (17.76, 33.82)	14.41 (9.16, 16.86)	**< 0.001**
TNF-α	13.98 (12.53,16.84)	9.60 (6.47,13.04)	**0.009**	15.89 (14.22, 17.78)	8.97 (7.94, 12.41)	**< 0.001**
TRAIL	35.54 (34.96,55.72)	28.38 (20.62, 33.46)	**0.002**	40.94 (35.11, 44.48)	32.11 (30.12, 38.04)	**0.019**
IL-1α	9.96 (8.44, 11.48)	4.23 (1.86, 4.92)	**< 0.001**	16.45 (11.86, 22.98)	5.51 (3.21, 7.47)	**< 0.001**
IL-1β	2.90 (2.17, 3.26)	1.88 (1.40, 3.69)	0.316	2.16 (1.59, 3.41)	2.10 (1.38, 2.97)	0.603
IL-1ra	164.78 (145.93, 185.71)	70.70 (55.80, 128.01)	**0.004**	173.64 (157.39, 185.69)	107.36 (80.87, 150.82)	**< 0.001**
IL-2Rα	99.66 (83.29, 122.15)	95.46 (76.73, 141.57)	0.857	76.25 (47.30, 115.92)	101.79 (67.09, 133.93)	0.268
IL-4	2.18 (2.01, 2.59)	0.35 (0.17, 0.51)	**< 0.001**	2.34 (1.94, 3.01)	0.52 (0.39, 1.42)	**< 0.001**
IL-6	0.91 (0.56, 1.63)	0.65 (0.16, 0.93)	0.246	1.39 (1.01,2.45)	0.91 (0.26, 1.25)	**0.021**
IL-8	4.75 (3.46, 6.61)	2.28 (1.57, 3.16)	**0.003**	6.04 (4.40, 7.19)	3.14 (2.15, 4.36)	**< 0.001**
IL-9	58.55 (51.44, 65.08)	29.50 (26.06, 32.90)	**0.001**	65.00 (53.56, 69.74)	30.86 (24.88, 41.36)	**< 0.001**
IL-13	3.70 (2.31, 3.92)	1.94 (1.14, 5.21)	0.440	2.79 (2.00, 4.69)	2.23 (1.59, 4.70)	0.314
IL-16	41.51 (40.09, 49.67)	32.48 (28.14, 53.88)	0.341	37.98 (22.49, 52.19)	32.30 (25.81, 41.78)	0.535
IL-17A	5.12 (4.50, 5.90)	2.47 (1.16, 3.73)	**0.013**	6.68 (6.05, 8.09)	3.90 (2.39, 5.12)	**< 0.001**
IL-18	41.61 (35.17, 57.57)	40.37 (30.12, 102.04)	0.857	33.54 (26.08, 42.52)	29.43 (24.75, 58.68)	0.801

*Abbreviation*: *T0* baseline, *Q25* 25% quantile, *Q75* 75% quantile, *TB* tuberculosis

The levels of 16 cytokines including Eotaxin, MCP-1(MCAF), SDF-1α, MIP-1β, G-CSF, VEGF-A, Basic FGF, IFN-γ, TNF-α, TRAIL, IL-1α, IL-1ra, IL-4, IL-8, IL-9 and IL-17A were significantly higher in active TB patients compared with non-TB controls in both untreated and treated participants at T0. No significant difference was found for the serum levels of the rest 22 cytokines no matter whether preventative treatment conducted.

### Performance of the 16 cytokines in predicting active TB development

To further evaluate the performance on predicting active TB, the AUCs were calculated among untreated participants at T0 (Table 3). Ten cytokines were found to have an AUC > 0.85 (Eotaxin = 0.95, Basic FGF = 0.96, G-CSF = 0.89, IFN-γ = 0.95, IL-1α = 0.92, IL-1ra = 0.85, IL-4 = 1.00, IL-8 = 0.86, IL-9 = 0.90 and TRAIL = 0.88) (Table 3).

**Table 3:** Predicting value of the baseline (T0) serum cytokines levels on active TB development among untreated controls

Cytokine	AUC (95%CI)	p for *z*-test	Cut-off (pg/ml)	Sensitivity,% (95% CI)	Specificity,% (95% CI)
Eotaxin	0.95 (0.87, 1.03)	**<0.001**	50.54	88.89 (51.75, 99.72)	94.44 (72.71, 99.86)
Basic FGF	0.96 (0.88, 1.02)	**<0.001**	17.49	88.89 (51.75, 99.72)	94.44 (72.71, 99.86)
G-CSF	0.89 (0.76, 1.02)	**0.001**	58.89	88.89 (51.75, 99.72)	88.89 (65.29, 98.62)
IFN-γ	0.95 (0.87, 1.03)	**<0.001**	17.41	88.89 (51.75, 99.72)	94.44 (72.71, 99.86)
IL-1α	0.92 (0.82, 1.02)	**0.001**	8.06	88.89 (51.75, 99.72)	88.89 (65.29, 98.62)
IL-1ra	0.85 (0.70, 1.00)	**0.003**	114.70	100.00 (66.37, 100.00)	72.22 (46.52, 90.31)
IL-4	1.00 (1.00, 1.00)	**<0.001**	1.16	100.00 (66.37,100.00)	100.00 (81.47,100.00)
IL-8	0.86 (0.73, 1.00)	**0.002**	3.44	77.78 (39.99, 97.19)	83.33 (58.58, 96.42)
IL-9	0.90 (0.77, 1.04)	**0.001**	38.85	88.89 (51.75, 99.72)	94.44 (72.71, 99.86)
IL-17A	0.80 (0.63, 0.98)	**<0.001**	3.48	100.00 (66.37, 100.00)	72.22 (46.52, 90.31)
MCP-1(MCAF)	0.79 (0.59, 0.99)	**0.016**	10.34	66.67 (29.93, 92.51)	83.33 (58.58, 96.42)
MIP-1β	0.75 (0.55, 0.94)	**0.040**	57.65	55.56 (21.20, 86.30)	94.44 (72.71, 99.86)
SDF-1α	0.79 (0.61, 0.97)	**0.016**	218.00	100.00 (66.37, 100.00)	50.00 (26.02, 73.98)
TNF-α	0.82 (0.66, 0.97)	**0.009**	10.48	100.00 (66.37, 100.00)	61.11 (35.75, 82.70)
TRAIL	0.88 (0.74, 1.01)	**0.002**	34.51	77.78 (39.99, 97.19)	88.89 (65.29, 98.62)
VEGF-A	0.83 (0.66, 1.00)	**0.006**	14.31	88.89 (51.75, 99.72)	72.22 (46.52, 90.31)

*Abbreviation*: *AUC* area under curve, *TB* tuberculosis

### Serum levels of the 16 selected cytokines in different time-points

In treated participants, as compared with level at T0, statistically significant reductions were found for serum levels of Eotaxin, SDF-1α, Basic FGF, IFN-γ, IL-1ra, IL-4 and IL-8 in active TB group at T1. For non-TB group, as compared with T0, there were statistically significant decreases in serum levels of Eotaxin, SDF-1α, TRAIL, IL-1ra and IL-8 while increases for MIP-1β and IL-9 (Table 4).

**Table 4:** Median serum levels of the selected cytokines at different time point in participants with and without preventive treatment respectively

^a^Cytokine	Active TB			Non-TB		
	T0	T1	p for Wilcoxon signed rank test	T0	T1	p for Wilcoxon signed rank test
Median value (Q25-Q75) (pg/ml) in treated participants (active TB, n = 12; non-TB, n = 24)						
Eotaxin	69.18 (58.08, 87.05)	45.18 (36.09,75.64)	**0.021**	37.14 (26.61, 66.58)	33.34 (22.22, 48.22)	**0.010**
MCP-1(MCAF)	14.08 (10.57, 20.05)	11.60 (9.68, 15.26)	0.052	7.91 (5.65, 10.10)	7.38 (5.01, 12.86)	0.300
MIP-1β	50.75 (47.05, 63.37)	56.63 (50.95, 61.09)	0.470	38.06 (30.83, 44.48)	49.03 (44.11, 54.68)	**< 0.001**
SDF-1α	309.99 (234.09, 339.68)	229.56 (203.70, 261.91)	**0.007**	211.88 (190.64, 251.72)	177.85 (158.87, 220.24)	**0.008**
G-CSF	89.49 (73.15, 97.55)	71.50 (60.18, 85.92)	0.110	49.40 (36.56, 62.44)	65.14 (39.89, 78.41)	0.040
VEGF-A	46.18 (38.72, 62.85)	42.22 (28.75, 52.27)	0.339	13.34 (3.37, 23.12)	6.43 (0.25, 37.39)	0.187
Basic FGF	23.62 (20.22, 25.25)	19.51 (18.79, 21.27)	**0.010**	11.60 (7.52, 17.13)	11.28 (6.52, 20.05)	0.459
IFN-γ	20.89 (17.76, 33.82)	19.74 (12.71, 26.54)	**0.021**	14.41 (9.16, 16.86)	13.87 (5.52, 16.82)	0.320
TNF-α	15.89 (14.22, 17.78)	15.41 (12.66, 17.78)	0.349	8.97 (7.94, 12.41)	10.36 (6.77, 13.98)	0.478
TRAIL	40.94 (35.11, 44.48)	36.59 (36.42, 43.32)	0.733	32.11 (30.12, 38.04)	31.28 (23.18, 37.34)	**0.037**
IL-1α	16.45 (11.86, 22.98)	13.01 (10.72, 14.53)	0.015	5.51 (3.21, 7.47)	5.24 (1.52, 14.53)	0.129
IL-1ra	173.64 (157.39, 185.69)	126.44 (114.19, 133.83)	**0.002**	107.36 (80.87, 150.82)	95.66 (55.77, 131.74)	**0.009**
IL-4	2.34 (1.94, 3.01)	1.61 (1.36, 2.01)	**0.013**	0.52 (0.39, 1.42)	0.64 (0.04, 1.55)	0.575
IL-8	6.04 (4.40, 7.19)	4.05 (3.58, 4.64)	**0.017**	3.14 (2.15, 4.36)	2.04 (0.70, 3.34)	**< 0.001**
IL-9	65.00 (53.56, 69.74)	63.83 (55.94, 77.15)	0.733	30.86 (24.88, 41.36)	37.00 (31.20, 64.25)	**< 0.001**
IL-17A	6.68 (6.05, 8.09)	5.74 (5.05, 6.21)	0.034	3.90 (2.39, 5.12)	2.97 (0.57, 5.90)	0.187
Median value (Q25-Q75) (pg/ml) in untreated controls (active TB, n = 9; non-TB, n = 18)						
Eotaxin	86.14 (56.31, 100.06)	55.38 (39.96, 59.73)	**0.012**	34.46 (29.40, 37.82)	28.43 (21.67, 38.27)	0.265
MCP-1(MCAF)	12.60 (9.20, 16.31)	12.17 (11.41, 14.12)	0.570	6.81 (5.14, 9.74)	6.88 (5.14, 10.16)	0.212
MIP-1β	48.10 (38.42, 57.29)	57.68 (49.97, 59.57)	0.301	38.03 (33.57, 43.83)	48.19 (35.40, 54.16)	**0.024**
SDF-1α	268.74 (253.62, 357.07)	223.75 (205.00, 256.94)	**0.039**	219.86 (181.17, 275.33)	175.13 (142.03, 225.55)	**0.027**
G-CSF	70.18 (68.86, 89.17)	78.74 (64.20, 94.98)	0.820	45.96 (39.89, 52.46)	52.17 (32.71, 62.19)	0.304
VEGF-A	30.46 (19.20, 52.30)	27.04 (14.41, 35.17)	0.496	10.62 (3.37, 15.07)	13.88 (0.25, 27.04)	0.426
Basic FGF	19.51 (19.51, 27.46)	19.51 (18.06, 20.92)	0.055	9.91 (7.71, 11.27)	11.98 (6.93, 15.78)	0.186
IFN-γ	26.08 (18.59,31.97)	20.47 (14.40,26.28)	0.055	9.48 (8.21, 14.39)	9.52 (4.75, 15.24)	0.702
TNF-α	13.98 (12.53,16.84)	14.46 (11.81,16.84)	0.688	9.60 (6.47,13.04)	9.00 (5.07, 11.08)	0.246
TRAIL	35.54 (34.96,55.72)	40.35 (38.76,42.93)	0.820	28.38 (20.62, 33.46)	25.86 (18.61, 32.15)	0.694
IL-1α	9.96 (8.44, 11.48)	11.48 (7.68, 14.53)	0.742	4.23 (1.86, 4.92)	4.32 (1.52,9.96)	0.096
IL-1ra	164.78 (145.93, 185.71)	116.49 (111.89, 129.64)	0.078	70.70 (55.80, 128.01)	89.36 (40.01, 109.54)	0.265
IL-4	2.18 (2.01, 2.59)	1.67 (1.42, 1.87)	**0.027**	0.35 (0.17, 0.51)	0.58 (0.05, 1.16)	0.072
IL-8	4.75 (3.46, 6.61)	3.81 (3.22, 4.05)	**0.047**	2.28 (1.57, 3.16)	1.91 (0.84, 2.39)	**0.030**
IL-9	58.55 (51.44, 65.08)	66.41 (58.89, 69.24)	0.496	29.50 (26.06, 32.90)	35.55 (23.97, 64.91)	**0.003**
IL-17A	5.12 (4.50, 5.90)	5.43 (4.50, 6.05)	0.578	2.47 (1.16, 3.73)	3.12 (1.16, 4.19)	0.832

*Abbreviation*: *T0* baseline, *T1* At the end of preventive treatment; *Q25* 25% quantile, *Q75* 75% quantile, *TB* tuberculosis

^a^ Those cytokines, which had a significantly higher level in active TB individuals compared with non-TB controls at T0 were selected

In untreated participants, as compared with level at T0, statistically significant reductions in serum levels of Eotaxin, SDF-1α, IL-4 and IL-8 were found in active TB group at T1. For non-TB group, as compared with T0, there were statistically significant decreases in serum levels of SDF-1α and IL-8 while increases for MIP-1β and IL-9 (Table 4).

**Fig. 1:**
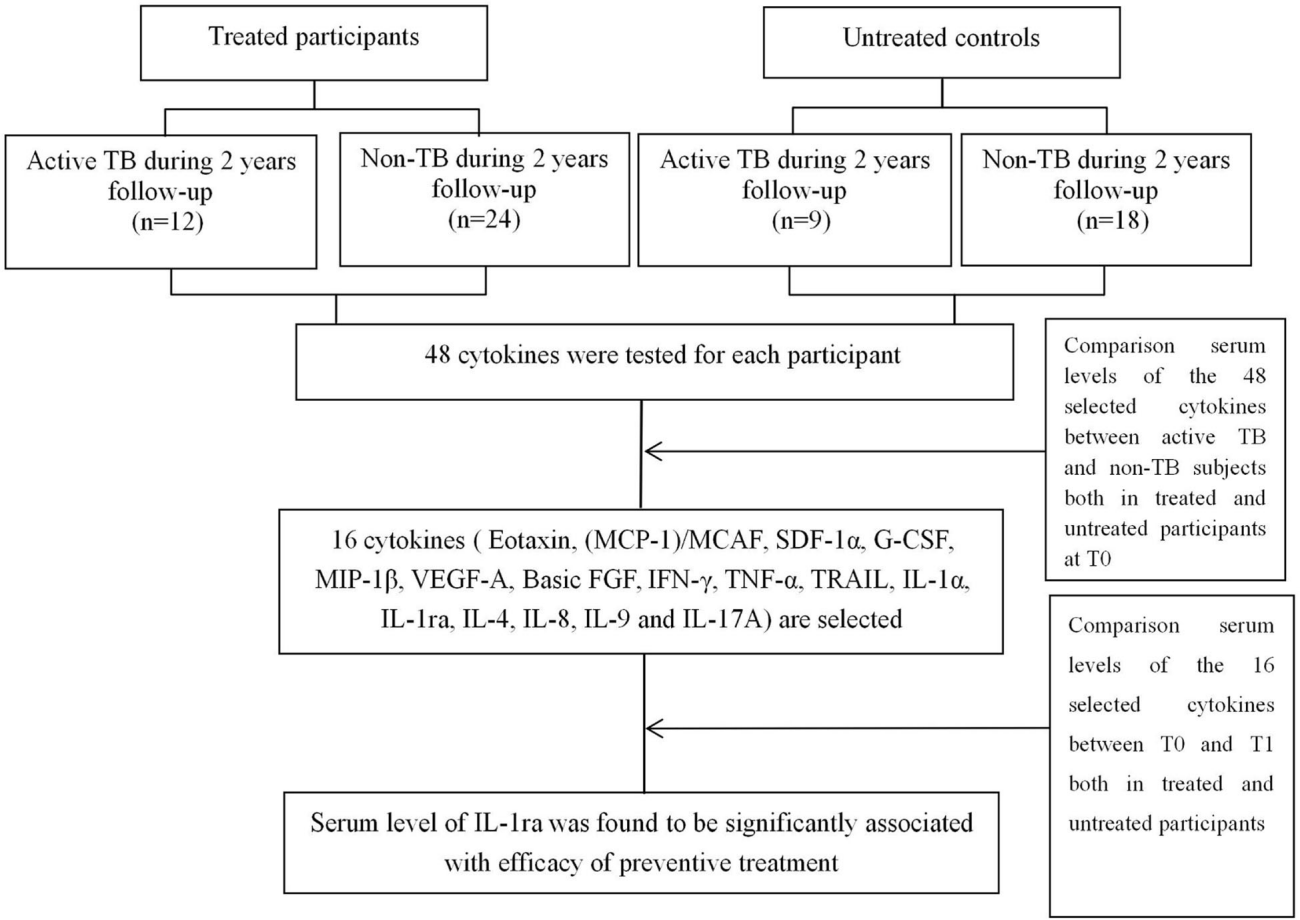
Flow chart to identify the relation between serum cytokines level and preventive treatment efficacy. A total of 12 active TB cases and 24 non-TB individuals from treated participants as well as 9 active TB cases and 18 non-TB individuals from untreated controls, paired by gender and age, were included in the present study. The levels of 16 cytokines were significantly higher in active TB cases as compared with non-TB controls both in treated participants and untreated controls at T0. Only the level of IL-1ra declined significantly from T0 to T1 in treated participants, but not in untreated controls

As shown in Fig. 1, only the median level of IL-1ra declined significantly from T0 (173.64 IU/ml) to T1 (126.44 IU/ml) in active TB group (*p* = 0.002) and from T0 (107.36 IU/ml) to T1 (95.66 IU/ml) in non-TB group (*p* = 0.009) among treated participants. No statistically significant difference was found for IL-1ra level at T1 as compared with T0 both in active TB and non-TB groups among untreated participants (*p* = 0.078 for active TB group, *p* = 0.265 for non-TB group) (Table 4).

## Discussion

Our results provided new insight into using serum cytokines as biomarkers to evaluate host responses to LTBI preventative treatment. The baseline serum levels of 16 cytokines were found to be significantly higher in those developed active TB laterly as compared with non-TB controls in both untreated and treated participants. Among them, in particular, IL-1ra responses declined significantly after treatment in treated participants, suggesting a predisposing role as biomarker to predict the development of active TB.

To our knowledge, only a handful of studies ever explored the relationship between cytokines and TB development. A study from Amsterdam cohort reported that the expression of IL-13 could predict the development of TB within months before the onset of clinical symptoms among HIV-infected individuals [16–18]. Another prospective study found that IP-10 levels in active patients were higher than in household and community controls [19]. A subsequent study conducted in participants with HIV-TB and active TB made similar observations, which reported that blood IP-10 levels were significantly higher in active TB patients than in controls, regardless of HIV infection [20]. Such previous findings were not observed in our study, different demographic and clinical characteristics of the study participants and different time points selected for cytokine measurements might result in the inconsistence. As most of the previous studies compared the level of cytokines between active TB patients and LTBI participants through case-control design, the influence of individual difference within group on the cytokines response could not be excluded [21–25]. Our longitudinal cohort-based study provides more solid evidence using dynamic changes of the serum cytokines in the same person. However, study with large samples size are needed to verify the current result.

Our study showed that the level of IL-1ra declined significantly during therapy both in active TB and non-TB groups. But such a difference was not observed for untreated participants. It was consistent with a prospective study which observed a statistically significant decline in IL-1ra responses at 6 and 9 months after treated with isoniazid for 9 months in participants with LTBI [12]. Our results showed the decline occurred as early as 1 week after the treatment, which suggested a possibility that IL-1ra level might be used for real-time treatment effect monitoring. IL-1ra is a cytokine produced by monocytes, macrophages, and dendritic cells, which prevents the binding of IL-1α as well as IL-1β to IL-1R1 by competitively blocking IL-1RI receptors [26, 27]. IL-1ra expression played an important role during mycobacterial antigen-elicited granuloma formation, an immune and physical barrier to contain the infection and prevent MTB dissemination [28, 29]. A prospective study conducted in active TB cases found that serum IL-1ra concentrations were significantly reduced after anti-TB treatment and the level of IL-1ra was higher in patients with delayed treatment response than those with good response to therapy [30]. These studies consistently suggested that IL-1ra might be potential correlates of successful treatment in both active TB and LTBI individuals. Our results provide novel insight into using IL-1ra as an early prognosis biomarker to evaluate the performance of the preventive treatment. Besides, it was recently reported that *M.tb*-infected mice receiving anti-IL-1ra antibody had significantly lower bacterial burdens in their lungs as compared to PBS-treated controls [31]. Additionally, patients with rheumatic diseases receiving monoclonal IL-1ra antibodies had a significantly increased risk of TB re-activation [32]. These findings provide further evidence to support the relation between higher levels of baseline IL-1ra and the risk of developing active TB observed in our study. However, cytokine measurements were not repeated at the time of diagnosis of active TB in our study, whether serum level of IL-1ra could differentiate active TB and LTBI needs to be further verified.

When interpreting the results of the study, some limitations should be kept in mind. First, although we used the same Luminex kit to test serum samples collected at different time points, the influence of storage time on sample quality could not be completely ruled out. Second, considering the feasibility of monitoring peripheral blood cytokines after preventive treatment un-stimulated serum samples were measured in our study. Therefore, the dynamic change of cytokine concentrations observed in our study might could not accurately reflect the changes in immune levels caused by TB-specific pathogens. Our results needs to be verified by further study with larger sample size and with specific antigen stimulated samples [33]. Third, due to the limited sample size, we pooled the treated participants who were treated with different regimens for analysis, which might have an impact on the outcomes. Fourth, as the RCT targeting elder population with attenuation of immunity, generalization of the study results should be cautious. Fifth, in order to avoid potential confounding effect, controls not developed active TB were matched with cases by age and gender, but bias caused by other potential confounders could not be excluded in our study [6].

## Conclusions

Our results suggested that serum level of IL-1ra decreased along with preventive treatment and might be used to predict disease progression. It provided a clue for exploring prognosis biomarkers to evaluate the performance of LTBI treatment, but our findings needs further verification by studies with larger sample size in different populations.

## Data Availability

The datasets used and/or analysed during the current study are available from the corresponding author on reasonable request.

## Abbreviations

LTBI: Latent tuberculosis infection
TB: Tuberculosis
*M.tb*: *Mycobacterium tuberculosis*
TST: Tuberculin skin test
IGRAs: Interferon-γ Release Assays
QFT: QuantiFERON TB Gold In-Tube
IP-10: Interferon-inducible protein − 10
MCP-1: Macrophage chemoattractant protein-1
MIP-1α: Macrophage inflammatory protein-1α
GM-CSF: Granulocyte macrophage colony stimulating factor
ROC: Receiver operating characteristic
AUCs: Area under ROC curves
